# Tubeless Ureteroscopy: A Comprehensive Literature Review

**DOI:** 10.7759/cureus.109232

**Published:** 2026-05-19

**Authors:** Ahmed S Aboelatta

**Affiliations:** 1 Urology Department, Chesterfield Royal Hospital NHS Foundation Trust, Calow, GBR

**Keywords:** double-j stent, laser lithotripsy, lower urinary tract symptoms, temporary ureteral stent, tubeless ureteroscopy

## Abstract

Stent omission post-ureteroscopy, defined as performing ureteroscopic stone treatment without placement of a postoperative ureteral stent, has evolved from an experimental concept to a recognized, guideline-endorsed practice for appropriately selected patients. A persistent gap exists between the available evidence and clinical adoption, with ureteral stent placement remaining the predominant practice following ureteroscopic procedures at most centres worldwide, reflecting ongoing tension between clinical caution and evidence-based care. This comprehensive literature review synthesizes evidence from randomized controlled trials, systematic reviews, meta-analyses, registry studies, and prospective cohort studies examining the safety, efficacy, patient-reported outcomes, healthcare utilization, and patient selection criteria for tubeless ureteroscopy published through February 2026. Key findings consistently demonstrate that stent omission in uncomplicated cases reduces lower urinary tract symptoms, hematuria, and stent-related morbidity without meaningfully increasing the risk of postoperative complications or unplanned healthcare encounters. Evolving technologies, including laser dusting techniques, high-powered holmium and thulium fiber lasers, suction ureteral access sheaths, and single-use flexible ureteroscopes, have further expanded the candidate pool for stentless approaches. Landmark trials such as the SOUL study and a 2025 multicenter randomized controlled trial provide the most rigorous modern evidence supporting stent omission. Tubeless ureteroscopy is safe, superior for patient experience, and warranting of broader adoption in appropriately selected patients. Current evidence supports American Urological Association and European Association of Urology guideline recommendations for stent omission in uncomplicated cases.

## Introduction and background

Ureteroscopy (URS) combined with laser lithotripsy is one of the most commonly performed surgical procedures for the management of nephrolithiasis and ureterolithiasis in the United Kingdom and across the developed world [1,2]. Utilization of URS has grown substantially over the past two decades, driven by improvements in endoscope design, digital optics, laser technology, and miniaturization of working tools [3]. The procedure is associated with high stone-free rates, a favorable safety profile, and suitability for outpatient or ambulatory surgery settings [4].

At the conclusion of ureteroscopic stone treatment, the urologist faces a fundamental decision: whether to place a temporary ureteral stent (double-J stent) or leave the patient tubeless - that is, without any indwelling ureteral drainage. The rationale for stent placement has historically centered on ensuring ureteral patency, preventing obstruction from edema or residual stone fragments, and facilitating drainage of the renal unit during healing [5]. However, ureteral stents are a well-recognized source of patient morbidity, commonly causing lower urinary tract symptoms (LUTS), flank pain, hematuria, frequency, urgency, and dysuria, and often necessitating a subsequent procedure for removal [6].

Tubeless URS refers to the deliberate omission of ureteral stent placement following an uncomplicated ureteroscopic procedure. This approach has gained considerable traction over the past two decades, evolving from early observational studies to guideline-supported practice. Both the American Urological Association (AUA) and the European Association of Urology (EAU) now endorse selective stent omission in appropriately chosen patients following uncomplicated URS [3].

## Review

Review of literature 

Historical Context and Early Studies 

The first prospective investigations into stent omission emerged in the late 1990s and early 2000s. One of the earliest and most influential studies was conducted by Hollenbeck et al. [7], who analyzed 837 ureteroscopic procedures performed between January 1997 and January 2002 at a single institution. Stents were not placed in 226 cases (32%). The overall postoperative complication rate was 18% in the stentless group, with obstructive complications occurring in 12%. Multivariate logistic regression identified multiple risk factors associated with postoperative morbidity in unstented patients, including renal pelvic stone location (p=0.02), performance of lithotripsy (p=0.03), bilateral procedures (p=0.007), history of urolithiasis (p<0.0001), diabetes mellitus (p=0.06), recent or recurrent infection (p<0.0001), and operative time of 45 minutes or greater (p=0.07). This foundational study established the evidence base for selective stent omission and proposed criteria for identifying suitable candidates that continue to inform modern practice.

A complementary body of observational and retrospective literature from the early 2000s focused on defining the clinical spectrum of post-URS complications in the stentless setting. Several groups reported that the most common complications after stentless URS were self-limiting obstructive episodes and urinary tract infections, with very few patients requiring emergent intervention. These data began to inform a growing clinical consensus that routine stenting in all cases was not justified and that a risk-stratified approach was both feasible and appropriate. Borboroglu et al. [8] published a pivotal multi-institutional prospective randomized controlled trial comparing stented versus unstented patients after URS for distal ureteral calculi, demonstrating comparable pain scores, complication rates, and unplanned medical visit rates between groups in uncomplicated cases. Cheung et al. [9] similarly reported a prospective randomized investigation of outpatient holmium laser lithotripsy using miniscopes, finding that stent omission did not adversely affect postoperative outcomes when patient selection criteria were met. Denstedt et al. [10] contributed a rigorous prospective randomized trial directly comparing nonstented versus stented ureteroscopic lithotripsy, finding significantly lower urinary frequency, urgency, and dysuria in the stentless group with comparable serious complication rates, providing early Level 1 evidence for stent omission in appropriately selected patients.

Burden of Ureteral Stent-Related Morbidity 

A prerequisite for understanding the appeal of tubeless URS is recognizing the substantial morbidity attributable to ureteral stents. Joshi et al. [6] published foundational work characterizing the quality of life (QoL) impact of indwelling ureteral stents using the Ureteral Stent Symptom Questionnaire (USSQ), demonstrating that the majority of stented patients experience pain (80%), urinary symptoms (90%), and impairment of daily activities (32%) during the period of indwelling stent. This study established the USSQ as a validated instrument for quantifying stent-related morbidity and underscored the extent to which ureteral stents adversely affect patient QoL. The STudy to Enhance uNderstanding of sTent-Associated Symptoms (STENTS) [11] was a landmark multicenter prospective observational cohort study that enrolled 424 patients undergoing URS with ureteral stent placement across four clinical centers. Participants completed standardized validated symptom instruments, including the Brief Pain Inventory (BPI), Patient-Reported Outcomes Measurement Information System (PROMIS) pain measures, the USSQ Urinary Score, and the Lower Urinary Tract Dysfunction Research Network Symptom Index-10 items (LURN SI-10), at baseline and on postoperative days one, three, and five, at stent removal, and 30 days post-removal. The STENTS study quantified stent-related symptoms over time and identified risk factors for increased stent-associated symptoms (SAS), finding that younger age, female sex, chronic pain conditions, and prior severe stent symptoms were significant predictors of worse stent-related morbidity. Importantly, ureteral access sheath (UAS) use was not independently associated with increased SAS after multivariable adjustment [11]. 

Supplementing these patient-reported outcome data, several health economic analyses have demonstrated that stent-related morbidity carries meaningful indirect costs. Lost work productivity, additional medical encounters for stent-related symptoms, and analgesic requirements all contribute to the economic burden of routine stenting. These considerations reinforce the clinical relevance of stent omission as not only a patient-centered intervention but also a potentially cost-saving one for healthcare systems. The introduction of tamsulosin and other alpha-adrenergic antagonists as pharmacological mitigation strategies for stent-related LUTS has provided some symptom relief, but has not eliminated the morbidity burden, further supporting the case for stent omission in suitable candidates. Deliveliotis et al. [12] demonstrated in a randomized controlled trial that alpha-1 blockers modestly attenuate stent-related LUTS but do not fully eliminate them, underscoring the ceiling effect of pharmacological palliation and reinforcing the clinical logic of stent avoidance as the most effective patient-centered strategy [12]. 

From a health economics perspective, Pengfei et al. [13] performed a systematic review and meta-analysis quantifying the cost implications of ureteral stenting after URS, concluding that stenting was associated with significantly higher direct costs principally attributable to the stent removal procedure, stent-related medical encounters, and analgesic requirements, with no demonstrable reduction in serious complication rates that would justify the additional expenditure in uncomplicated cases [13]. 

Systematic Reviews and Meta-Analyses 

The most methodologically rigorous systematic review was conducted by Ordonez et al. [14] and published in the Cochrane Database of Systematic Reviews. The updated review (2022) included 23 randomized controlled trials comprising 2,656 randomized patients. Key findings included the possibility that stenting may slightly reduce unplanned return visits (very low certainty of evidence), while pain on the day of surgery was probably similar between stented and unstented groups (moderate certainty of evidence). The Cochrane review concluded that existing evidence illustrated trade-offs between risks and benefits, with most findings based on low or very low certainty of evidence, and called for higher-quality trials to resolve remaining uncertainties [14]. 

Pais et al. [15] conducted a systematic review and meta-analysis that specifically evaluated the risk of unplanned medical visits as an outcome of stent omission. Seventeen studies comprising 1,943 participants met inclusion criteria. Unstented patients were significantly more likely to have an unplanned medical visit compared to those who received a post-URS stent (OR 1.63, 95% CI 1.15-2.30). However, the clinical magnitude of this association was small, and subsequent analyses have questioned the generalizability of this estimate given the heterogeneity of included studies and the predominantly historical nature of the data [15]. 

Earlier meta-analyses by Nabi et al. [5] found no clear benefit of stenting with respect to postoperative pain, infection, or stone-free rates in uncomplicated procedures. 

A 2011 meta-analysis by Pengfei et al. [13] pooled data from multiple randomized controlled trials and found that while stented patients had lower rates of ureteral colic and unplanned visits in the immediate postoperative period, they experienced significantly higher rates of urinary symptoms, dysuria, and hematuria throughout the period of stent indwelling. The net effect on patient satisfaction and overall QoL favored the stentless group in this analysis. Subsequent pooled analyses have consistently reinforced the finding that the choice between stenting and stent omission involves a trade-off between two distinct categories of morbidity: early obstructive or infectious risk versus the ongoing morbidity of an indwelling foreign body [13]. 

Taken together, the meta-analytic literature supports stent omission as the preferred strategy in uncomplicated cases, with the caveats that available evidence remains heterogeneous and that high-quality contemporary randomized data were limited until the publication of the Stent Omission after Ureteroscopy and Lithotripsy (SOUL) trial and the 2025 multicenter randomized controlled trial (RCT) which was designed to evaluate the QoL of patients undergoing ureteral stent omission compared to stent placement following uncomplicated ureteroscopic treatment of upper urinary tract stones [16]. 

Key Randomized Controlled Trials 

Bach et al. [17] conducted the Fast-Track Stent (FaST) study, a prospective randomized controlled trial that evaluated short-term external ureter stenting (stent on a string, removed at 24-48 hours) versus routine double-J stent placement. Short-term external stenting showed significant benefit in patient-reported outcomes compared to prolonged double-J stenting, supporting expedited recovery pathways and providing evidence that routine prolonged internal stenting is unnecessary in uncomplicated procedures. The FaST study established that the duration of stenting is an important determinant of stent-related morbidity: even when a stent was deemed necessary, early removal conferred substantial patient benefit relative to conventional indwelling periods [17].

Denstedt et al. [10] conducted one of the early landmark randomized controlled trials directly comparing stented versus non-stented ureteroscopic lithotripsy. In their prospective trial of 58 patients, those without stents experienced significantly less urinary frequency, urgency, and dysuria during the postoperative period, while rates of serious complications were comparable between groups. This trial provided early Level 1 evidence that stent omission was feasible and safe in appropriately selected patients and helped establish the foundation for subsequent larger-scale investigations [10].

Verhovsky et al. [18] examined tubeless ureterorenoscopy in the context of modern laser technology - specifically 120W holmium laser using a dusting technique. The retrospective analysis evaluated postoperative pain, complications, and readmissions after tubeless URS in patients treated with high-power laser dusting. The study found that tubeless URS using the dusting technique was safe, with acceptable complication and readmission rates, demonstrating that the combination of modern laser technology with stent omission was clinically viable. This study was among the first to systematically evaluate stent omission in the context of contemporary high-powered laser systems, bridging the gap between early feasibility data and modern practice [18].

Allam et al. [19] published a randomized controlled trial in 2023 comparing routine ureteral stenting versus stent omission following uncomplicated ureteroscopic treatment of upper ureteral and renal stones. The study enrolled 100 patients and found no significant difference in 30-day complication rates between groups (12% stented vs. 10% unstented; P=0.75), while stent omission was associated with significantly lower rates of LUTS, dysuria, and hematuria in the early postoperative period. This trial corroborated the findings of earlier randomized studies and provided contemporary evidence supporting stent omission in the modern laser lithotripsy era [19].

The most impactful recent trial was presented by Bechis et al. [20] at the 2025 AUA Annual Meeting in Las Vegas, Nevada. This prospective multicenter randomized controlled trial enrolled patients at six institutions. Among 102 patients with nonobstructing renal stones up to 1.5 cm who underwent URS, 74 eligible patients were randomized 1:1 to stent placement (n=36) or stent omission (n=38). Of the preliminary results, there was no significant difference in 30-day complications between groups (8.3% stented vs. 10.5% unstented; P=0.748) [20].

Stent omission shortened case time by an average of 6.5 minutes. Patient-reported pain (by PROMIS) was significantly improved in the stent omission group, with a 7-8 point score improvement exceeding the minimally important clinical difference. QoL showed a 16-19 point advantage in the stent omission group. Patients who underwent stent omission were also significantly more likely to indicate they would choose the same procedure again. The investigators concluded that stent omission after uncomplicated URS for renal stones ≤1.5 cm is safe, reduces operative time, substantially improves patient-reported pain and QoL, and should be considered standard practice [20].

Registry and Collaborative Studies 

The Michigan Urological Surgery Improvement Collaborative (MUSIC) Reducing Operative Complications From Kidney Stones (ROCKS) has produced the most comprehensive real-world registry data on stenting practices and outcomes. Hiller et al. [21] of 9662 ureteroscopies demonstrated using MUSIC registry data (2016-2019) that ureteral stent placement following URS significantly increases emergency department visits in this statewide surgical collaborative. This finding directly challenged the prevailing assumption that stenting reduces healthcare utilization and highlighted the paradox that a practice intended to minimize complications was itself generating a measurable burden of postoperative morbidity in the real-world setting [21]. 

Subsequently, Hiller et al. [22] developed appropriateness criteria using the RAND/UCLA Appropriateness Method (RAM), convening a panel of 15 urologists who identified seven key variables influencing stent decision-making: stone size, stone location, pre-stenting status, urinalysis/urine culture result, non-balloon ureteral dilation, use of UAS, and residual stone fragments. This structured decision-making framework represented an important advance in operationalizing the evidence base for clinical practice, providing urologists with a practical tool to identify appropriate candidates for stent omission rather than relying solely on individual clinical judgment [22]. 

DiBianco et al. [23] extended this work by examining outcomes specifically in pre-stented patients undergoing URS through the MUSIC/ROCKS registry. They found that pre-stented patients receiving a postoperative stent were more than twice as likely to have a postoperative emergency department visit or hospitalization compared to those with stent omission. This analysis established pre-stented patients as the highest-yield population for stent omission from a quality improvement standpoint, as their ureters have been passively dilated and their risk of postoperative obstruction is lowest, while their exposure to stent-related morbidity is highest when a second stent is placed after URS. 

Hamouche et al. [24] conducted a prospective case cohort study from the Registry for Stones of the Kidney and Ureter (ReSKU) after analyzing 470 consecutive patients undergoing URS (92 patients, 19.5%, were stentless). Stentless URS was associated with lower stone burden (p<0.001), pre-existing ureteral stent, absence of access sheath use, and shorter operative time (31 vs. 58 minutes, p<0.001). Postoperative gross hematuria and LUTS were significantly less common in stentless patients (p=0.02 and p=0.01, respectively). No difference in postoperative complications was observed between groups (15.2% vs. 12.0%, p=0.385), and no patient in the stentless group required emergent stent placement. The ReSKU analysis provided important real-world validation of stentless URS outcomes outside the controlled setting of a clinical trial. 

Additional registry data from the Clinical Research Office of the Endourological Society (CROES) URS global study [25] have corroborated these findings on an international scale, demonstrating wide variation in stenting practices across countries and practice settings, with stenting rates exceeding 80% in most regions. The CROES global study analyzed 11,885 patients undergoing URS at 114 institutions across 32 countries, providing the largest international dataset on ureteroscopic practice patterns and outcomes to date. The CROES data highlighted that pre-stenting status, stone location, and operative complications were the most consistent determinants of the decision to place a postoperative stent, though a substantial proportion of stent placements occurred in patients who would have been considered appropriate candidates for stent omission by contemporary criteria. These registry data underscore that the evidence-practice gap in stent omission is a global phenomenon, not limited to specific healthcare systems or regions, and highlight the urgent need for international quality improvement initiatives to align stenting practices with contemporary evidence [25]. 

The SOUL Trial 

The SOUL trial [16] is a landmark pragmatic multicenter combined randomized and observational clinical trial coordinated through MUSIC and funded by the Patient-Centered Outcomes Research Institute (PCORI). The study employs an innovative combined design in which patients declining randomization are enrolled in an observational cohort, thereby capturing real-world outcomes across the full spectrum of patients presenting for uncomplicated URS. Study objectives include comprehensive assessment of health-related QoL, patient-reported outcomes including pain and urinary symptoms, time off work or school, and 30-day unplanned healthcare utilization. The SOUL trial enrolled approximately 800 patients across MUSIC-affiliated sites encompassing academic and community centers, private practices, and rural settings, thereby maximizing the external validity of findings across diverse practice environments. The trial's pragmatic design, which permits clinicians to exercise judgment regarding access sheath use and laser technique, reflects real-world clinical practice more faithfully than highly controlled efficacy trials. Preliminary results presented at AUA 2025 indicated no significant difference in 30-day complications between groups, with stent omission yielding superior patient-reported outcomes on validated instruments. The full publication of SOUL trial data, including long-term follow-up and comprehensive subgroup analyses, is anticipated to provide the most robust contemporary evidence base for stent omission. The SOUL trial results will also address remaining questions about stent omission in specific subgroups, including patients who undergo access sheath-assisted URS and those with stones in challenging anatomical locations [16].

Patient Selection Criteria and Clinical Guidelines 

The American Urological Association Surgical Management of Stones Guideline (2016, reaffirmed 2022, updated 2025) [3] recommends that ureteral stent omission may be offered to patients meeting all of the following criteria: normal contralateral kidney, no renal functional impairment, renal stone burden <1.5 cm, no planned second-stage URS, no ureteral injury or stricture, and no anatomical impediments to stone fragment clearance.

The 2025 AUA guideline update additionally notes that stent omission may be appropriate even when UASs have been used, informed by emerging trial data including the Bechis et al. multicenter RCT [20].

The EAU urolithiasis guidelines [26] align broadly with AUA criteria, supporting stent omission in uncomplicated ureteroscopic procedures with appropriate patient selection. The EAU 2024 Guidelines on Urolithiasis recommend stent omission as an acceptable and preferred strategy in patients without intraoperative complications, ureteral injury, significant residual stone burden, solitary kidney, or renal insufficiency. The EAU guidelines additionally emphasize the importance of surgeon experience and institutional case volume as contextual factors in the stent omission decision, recognizing that the safety of stentless URS is partly dependent on the ability to recognize and respond to intraoperative complications that might necessitate stent placement.

Both guidelines acknowledge that the evidence base, while substantially strengthened by recent trials, is still evolving with respect to specific subpopulations and technological contexts [3,26].

The MUSIC-developed appropriateness criteria operationalized the seven key variables identified by Hiller et al. into a practical decision support tool, with pre-stented patients representing the highest-yield group for stent omission, as their ureters have been passively dilated and their risk of postoperative obstruction is lowest [22].

The appropriateness criteria framework has been prospectively validated in the MUSIC registry population and has been disseminated through quality improvement initiatives targeting urologists with high routine stenting rates. Implementation of these criteria has been associated with meaningful reductions in postoperative stenting rates at participating sites without adverse effects on clinical outcomes [21].

The AUA guideline's extension of stent omission criteria to renal stones up to 1.5 cm, and the corroborating evidence from the Bechis et al. [20] RCT, marks a significant shift in the accepted scope of tubeless URS.

Impact of Modern Technologies 

The advent of high-powered holmium laser systems (80-120W) and the refinement of the dusting technique, in which stones are vaporized into submillimeter dust rather than fragmented into basketable pieces, has significantly expanded the candidate pool for stent omission [18]. Dusting minimizes the risk of significant residual stone fragments, reduces the need for UASs, causes less ureteral trauma, and creates conditions most favorable for safe stent omission. High-powered holmium laser systems also permit more efficient stone ablation with shorter operative times, which is an independent factor associated with lower rates of post-URS complications [18]. 

Thulium fiber lasers (TFL) represent the recent advancement in laser lithotripsy technology, offering stone ablation efficiency, reduced retropulsion, and effective dusting at lower energy settings compared to conventional holmium laser systems [27,28]. The TFL's continuous-wave emission at a wavelength of 1.94 μm confers superior water absorption and thus highly efficient stone vaporization, producing finer dust particles with less mechanical impact on the ureteral wall. An in vitro study [29] provided a comprehensive early review of TFL technology demonstrating that the TFL achieves stone vaporization at significantly lower pulse energies than holmium laser, with effective ablation at settings of 0.025 J and frequencies up to 2,000 Hz, generating mean particle sizes below 100 μm that are passable without basketing and that may spontaneously pass without significant obstruction risk. 

Early clinical studies [27,28] of TFL for ureteroscopic lithotripsy have demonstrated high stone-free rates and favorable safety profiles, and emerging data suggest that TFL-assisted dusting may further expand the pool of patients for whom stent omission is appropriate, as the finer and more homogeneous dust produced by TFL may be less likely to form obstructing aggregates in the collecting system. However, prospective data specifically examining stent omission outcomes in the TFL context are limited and represent an important priority for future research. 

Single-use flexible ureteroscopes have emerged as an important technological advance that has affected both the safety and accessibility of URS. These devices eliminate the downtime and repair costs associated with reusable scopes, provide consistent optical performance, and reduce cross-contamination risk. Somani et al. [30] conducted a comparative study demonstrating broadly equivalent stone-free and complication rates between digital and conventional flexible ureteroscopes, establishing that the quality of optics and instrument performance is adequate for complete stone clearance in either platform. From the perspective of stent omission, single-use scopes have facilitated the broader adoption of flexible URS in settings where reusable scope availability was previously a limiting factor, thereby expanding the population of patients who can benefit from minimally invasive stone treatment and potential stent omission [30]. 

The standardized and consistently functional optics and deflection angles of single-use scopes may additionally support more complete intrarenal stone visualization and clearance, which may in turn support safe stent omission by ensuring surgeons can confirm satisfactory stone dust dispensal before concluding the procedure [31]. 

The emergence of suction UASs - including flexible and navigable suction UAS (FANS) - maintains lower intrarenal pressures, improves stone clearance through active aspiration, and reduces the risk of pyelovenous backflow and postoperative sepsis. Tzelves et al. [32] conducted a systematic review and meta-analysis of 16 comparative studies, finding that suction significantly improved stone-free rates and reduced complication rates compared to standard URS. This technology addresses one of the historical concerns about stentless URS - namely, the potential for residual fragments to cause obstruction - by actively evacuating stone debris during the procedure, thereby reducing the likelihood of postoperative complications attributable to fragment-related obstruction. 

The STENTS study [11] demonstrated that UAS use was not independently associated with increased SAS after multivariable adjustment, and the Bechis et al. 2025 multicenter RCT [20] included UAS use without mandating postoperative stenting, with favorable outcomes in the stent omission group. 

There was a randomized controlled trial [33] comparing flexible versus traditional UASs in terms of intrarenal pressure and complications, finding that the flexible UAS (f-UAS) group demonstrated significantly higher stone-free rate (76.3% vs. 7.2%; P < 0.001) at one day postoperatively and a higher clearance rate of stone volume (98.11% vs. 91.78%; P < 0.001). The f-UAS group also had a lower total complications rate (9.9% vs. 22.4%; P = 0.003), lower incidence of fever (5.9% vs 11.9%; P = 0.001), shorter operative times (56.5 min vs. 59.9 min; P = 0.047), and lower usage rate of baskets (17.1% vs. 100%; P < 0.001) [33]. 

As UAS technology evolves toward smaller profiles, improved compliance, and integrated suction capabilities, the physiological rationale for mandatory post-procedure stenting becomes increasingly attenuated, further widening the pool of patients in whom stent omission can be practiced safely. The interaction between UAS use, infectious complications, and stent omission has emerged as a particularly important area of inquiry. Postoperative fever and urosepsis after URS are multifactorial in etiology and are primarily driven by intrarenal pressure elevation, pre-existing bacteriuria, and the presence of infected stone material, rather than by the absence of a ureteral stent per se. Tzelves et al. [32] demonstrated in their systematic review and meta-analysis that suction UAS significantly reduced both infectious complication rates and intrarenal pressure compared to standard URS without suction, suggesting that suction UAS may mitigate one of the principal residual risks cited in support of routine stenting. These data collectively support a paradigm in which UAS use, particularly suction-enabled UAS, may enhance the safety profile of stent omission by controlling the intraoperative conditions that drive postoperative infectious complications, rather than being a factor that necessitates stenting [11,20,32,33]. 

Discussion 

The body of evidence synthesized in this review demonstrates that tubeless URS represents a significant advancement in patient-centered urological care. The evolution from early observational studies to rigorous randomized controlled trials and large-scale registry analyses has established a clear benefit-risk profile favoring stent omission in appropriately selected patients. The burden of stent-related morbidity is substantial and well-documented across multiple validated patient-reported outcome measures. The STENTS study provided granular longitudinal data demonstrating that SAS peak in the early postoperative period and persist through removal, affecting QoL, work productivity, and patient satisfaction. 

This morbidity burden provides the primary rationale for stent omission when it can be performed safely, and it underscores the importance of distinguishing between the morbidity of stent omission and the morbidity of routine stenting when counseling patients about their options. The safety profile of stent omission is well-established. 

The Cochrane systematic review, the largest and most methodologically rigorous synthesis of randomized trial data, found no clinically meaningful increase in serious complications with stent omission. While early meta-analyses suggested a potential increase in unplanned medical visits, subsequent real-world registry data from MUSIC demonstrated the opposite effect-that stenting increased emergency department utilization. This discrepancy likely reflects differences between controlled trial settings and routine clinical practice and underscores the value of pragmatic registry studies in informing clinical decision-making. 

The 2025 multicenter randomized trial by Bechis et al. [20] represents a watershed moment for the field. This study employed rigorous methodological standards - intraoperative randomization, blinded allocation, validated patient-reported outcome measures, and multicenter enrollment - while addressing key limitations of prior trials. The finding that stent omission produced clinically meaningful improvements in pain and QoL without increasing complications provides the strongest modern evidence supporting practice change. 

Modern ureteroscopic technologies have expanded the feasible candidate pool for stent omission. High-powered laser dusting techniques minimize residual fragments, suction UASs reduce intrarenal pressure and infection risk, and improved flexible ureteroscopes enable more complete stone clearance with less ureteral trauma. These technological advances create favorable conditions for safe stent omission that were not present during early feasibility studies and contribute to the progressive shift in clinical practice toward stentless approaches. 

Several important limitations of the existing literature deserve acknowledgment. Most randomized trials have excluded complex cases, including patients with solitary kidneys, significant renal insufficiency, large stone burdens requiring staged procedures, ureteral strictures, and pregnant patients. Stent omission in these populations remains understudied, and clinical guidance for these scenarios relies largely on expert consensus and observational data. 

Long-term outcomes, including ureteral stricture rates, require follow-up beyond the typical 30-90-day study period, and the literature contains limited data on the incidence of delayed complications attributable to stent omission. Additionally, optimal patient selection criteria remain incompletely defined, and validated clinical prediction tools to identify ideal candidates for stent omission are still under development. The interaction between stent omission and specific technologies such as suction UASs and TFL requires a dedicated prospective study. 

A further limitation is that the large majority of available trial data originate from high-volume academic or tertiary referral centers with specialized endourology expertise, which may limit generalizability to community urologists or surgeons earlier in their URS learning curve. Implementation research examining how quality improvement initiatives, such as the MUSIC appropriateness criteria framework, translate stent omission evidence into real-world practice change across diverse practice settings is an important emerging area of inquiry. 

Future research priorities in tubeless URS encompass several clinically important dimensions. First, dedicated randomized controlled trials specifically enrolling patients with more complex anatomical features, including horseshoe kidneys, ureteropelvic junction obstruction, and prior endourological interventions, are needed to define the safety boundaries of stent omission beyond the uncomplicated stone patient. Second, patient-reported outcome instruments specifically validated for the tubeless context, capturing both the morbidity of stent omission (such as post-procedure pain and early obstructive episodes) and the morbidity prevented by avoiding stenting, would enhance the precision of comparative effectiveness research. Third, prospective studies examining the interaction of TFL technology and stent omission are needed, as TFL's unique ablation characteristics may alter the postoperative stone dust clearance dynamics in ways that are clinically relevant to stent omission safety. Fourth, machine-learning-based clinical prediction models integrating multiple patient and procedural variables into a validated stent omission suitability score would provide urologists with a practical, evidence-based tool at the point of care, moving beyond qualitative appropriateness criteria toward personalized decision support. Finally, global implementation science research examining the barriers to stent omission uptake, including surgeon training, medicolegal concerns, and institutional culture, is essential to translating the existing evidence base into meaningful reductions in unnecessary stent placement worldwide. 

## Conclusions

The over-all of evidences support stent omission after URS is safe in appropriately selected patients, with no meaningful increase in serious postoperative complications, hospital admissions, or clinically relevant obstruction across multiple randomised trials and large-scale registry studies. Patient-reported outcomes are substantially better without a stent, with clinically meaningful improvements in pain, urinary symptoms, and QoL consistently demonstrated across multiple validated instruments and study designs.
